# Supplementary material for: Are carbon emissions trading and green financial instruments synergistic? -Comprehensive quantitative research based on content analysis
Source: PLoS One. 2024 Mar 7;19(3):e0298601. doi: 10.1371/journal.pone.0298601 (PMC10919601; doi:10.1371/journal.pone.0298601)
Supplement: S1 Data — (ZIP) [file pone.0298601.s001.zip › Data/地级市Z_F工作报告.zip!/╜¡╬≈/╕o╓▌╩╨/└┤╫╘╣1⁄2╓┌║┼í╛┬φ┐╦╩2╛▌═°í┐_2012.docx]

各位代表：

　　现在，我代表市人民政府向大会报告工作，请予审议，并请各位市政协委员和列席会议的同志提出意见。

　　一、本届政府工作的回顾

　　五年来，在省委、省政府和市委的正确领导下，在市人大、市政协的监督支持下，市人民政府团结带领全市人民，深入贯彻落实科学发展观，积极响应省委、省政府“科学发展、进位赶超、绿色崛起”的号召，大力实施赶超发展战略，完成了市二届人大各次会议确定的主要任务，经济社会发展取得了令人瞩目的成就。

　　——综合实力逐步增强。全市生产总值从2005年的262亿元上升到2010年的630亿元，年均增长14.1%；财政总收入从15.37亿元上升到74亿元，年均增长36.9%；地方财政收入从10.2亿元上升到55.43亿元，年均增长40.3%；全社会固定资产投资从123亿元上升到648亿元，年均增长39.4%；社会消费品零售总额从102.7亿元上升到230.7亿元，年均增长17.6%。

　　——经济结构持续优化。三次产业比从2005年的27∶40∶33 调整到2010年的19∶49.9∶31.1，非农产业比重提高8个百分点。工业主导力量加强。规模以上工业增加值从37.14亿元上升到160亿元，年均增长25.8%。工业增加值占GDP的比重由27.6%提高到40.4%。初步形成了生物医药、纺织服装、机械制造、食品加工、化工建材、有色金属加工等六大主导产业。农业产业化水平提高。粮食产能增加6.28亿斤，单产提高36公斤。形成了南丰蜜桔、广昌白莲、东乡瘦肉型猪和崇仁麻鸡等一批特色农业产业。市级以上农业产业化龙头企业达302家，完成销售收入101.6亿元。第三产业活力增强。旅游收入年均增长超过20%。相继引进九江银行、上饶银行进驻抚州，启动了抚州农村商业银行和村镇银行的组建工作。

　　——发展基础不断夯实。向莆铁路开工建设；鹰瑞高速建成通车；抚吉高速和杭南长客专抚州段开工建设。全市高速公路通车里程达到390公里。实现了100%市至县通二级以上公路，100%的乡镇和行政村通油路或水泥路。500千伏变电站投入运行。抚州电厂获国家能源局批复同意开展前期工作。基本完成了全长113公里的抚河唱凯堤、中洲堤除险加固工程；完成了廖坊灌区一期工程。

　　——城乡面貌明显改观。五年来，全市共开工建设城市基础设施重点项目550个，完成投资430亿元。其中，市中心城区开工建设110个重点项目，完工或基本完工69个。实施新农村建设点3210个，投入资金17.5亿元。截至2010年底，全市城镇化率比2005年提高了10.6个百分点。市中心城区建成区面积扩大到50.2平方公里，人口增加到51.4万人，人均公园绿地面积达到16.6平方米，城市绿化率达到43.4%。

　　——改革开放深入推进。实施了新一轮32户国有工业企业改制，安置职工12344人；基本完成了省调度的非工口七个系统101户国企改制任务，安置职工15539人；完成了28个国有林场和森工企业改制，安置职工4719人。稳步推进投融资体制改革。发行了8亿元投资公司企业债券；建立健全了财政信用担保、中小企业融资担保和小额贷款担保三大信用担保体系，成立了47家担保机构，落实担保资本金6.8亿元，累计担保贷款总额达40.8亿元。推行医药卫生体制改革，群众就医费用明显降低、就医服务有效改善。推进林业产权配套制度改革，开展了商品林政策性保险的统保试点工作。不断扩大对外开放。2010年，全市实际利用外资首次突破2亿美元，年均增长14.6%；外贸出口首次突破5亿美元，年均增长46.8%。

　　——民生保障日益完善。五年来，民生方面的投入以年均46.1%的幅度递增。大力推进社会保险扩面征缴，不断提高保障水平。基本形成了覆盖全市各种经济形式从业人员、惠及各类群体的养老保险和医疗保险体系；城乡低保覆盖面提高了1.6个百分点，城乡低保标准分别提高到300元和130元。扎实推进保障性住房建设。开工建设各类保障性住房2.4万套、142.3万平方米，发放住房租赁补贴6800万元，解决了4.5万户中低收入家庭的住房困难。全面推行新型农村合作医疗制度，参合农民达280万，参合率达98%。千方百计扩大就业，着力改善群众生活。全市城镇年均新增就业再就业5.85万人、转移农村劳动力6.23万人。2010年，城市居民人均可支配收入达到14445元，农民人均纯收入达到5848元，分别是2005年的1.7倍和1.8倍。

　　——社会事业全面进步。教育事业整体推进。义务教育质量和水平不断提高，高考成绩连年居于全省前列，职业教育规模迅速扩大，高等教育实力得到增强。医疗卫生服务体系不断完善，基本实现了小病不出村、常见病不出乡、大病不出市县的目标。应对重大自然灾害及突发事件能力显著提升，夺取了抗冰救灾和抗洪救灾的重大胜利。深入开展平安创建活动和严打整治行动，社会治安综合治理得到加强。严格落实安全生产责任制和责任追究制，安全生产总体形势持续好转。扎实推进造林绿化“一大四小”工程，全市森林覆盖率提高3.4个百分点，达到64.5%。科技、文化、体育、人口和计划生育、食品药品监督、广播电影电视、新闻出版事业快速发展，扶贫移民、人民防空、民族宗教、外事侨务、涉台事务、统计、地震、气象、档案、史志、社会科学、红十字会和妇女儿童工作等各项事业有了新的进步。

　　——政府自身建设不断加强。认真开展学习实践科学发展观、民主评议政风行风、项目建设年、创业服务年和发展提升年等活动，切实改进机关作风，提升行政效能，清理和取消180项审批项目。加强对行政权力的监督，规范执法行为，自觉接受市人大及其常委会的法律监督、工作监督和市政协的民主监督，累计办理市人大代表建议854件，办理市政协提案1278件，满意率和基本满意率达98%以上。深化行政审批制度改革和公共资源交易制度改革，建立网上审批和电子监察系统，推行风险岗位廉能管理，强化经济责任审计和重点项目审计，有力推动了政府系统党风廉政建设和反腐败工作。

　　五年来的实践，给了我们许多重要启示：

　　一是必须始终坚持科学发展这个主题。坚持以经济建设为中心，紧紧扭住发展不放松；牢牢把握重要战略机遇期，推动经济社会跨越式发展；注重统筹发展，保持发展的协调性和可持续性；正确处理好改革发展稳定的关系，把改革力度、发展速度和社会可承受程度统一起来。

　　二是必须始终抓住项目建设这个关键。项目是抓手，项目是财源，项目是后劲，把发展的目标任务落实到一个个具体项目上，全力推进重大产业、重大基础设施和重大民生项目建设，以此推动经济社会全面进步。以是否能推动项目建设，作为检验各级干部能力和水平的重要标准。

　　三是必须始终把握改善民生这个目的。坚持发展为了人民、发展依靠人民、发展成果由人民共享；想问题、作决策、办事情，始终把群众利益放在首位，把实现好、维护好、发展好人民群众的根本利益作为政府工作的根本出发点和落脚点，以群众满意作为政府工作的根本标准。

　　四是必须始终维护团结和谐这个大局。领导班子的团结以及整个社会的和谐是加快发展的前提和基础。团结出生产力、凝聚力、战斗力，任何时候都把团结建立在民主集中制原则的基础上，建立在赶超发展的共同事业上，以团结聚合力，以团结求发展，以团结促和谐。

　　回顾过去的五年，成绩来之不易，经验弥足珍贵。这主要得益于省委、省政府和市委的正确领导，得益于市人大、市政协的监督支持，得益于历届政府、历任领导打下的良好基础，得益于社会各界的大力支持，得益于全市人民的团结奋斗。在此，我代表市人民政府，向所有为抚州改革发展稳定付出辛勤劳动、作出积极贡献的广大工人、农民、知识分子、干部职工，向驻抚部队指战员、武警消防官兵、公安干警，向热情支持和关心政府工作的人大代表、政协委员、各民主党派、工商联、无党派人士、各人民团体、中央和省驻抚各单位，向为抚州发展积极献计出力的老领导、老同志和海内外友好人士，向来抚州投资兴业、为抚州经济社会发展作出贡献的客商朋友，向所有关心、支持和帮助抚州发展的同志们、朋友们，表示衷心的感谢和崇高的敬意！

　　在肯定成绩的同时，我们必须清醒地看到面临的困难和问题，主要是：经济总量小、人均水平低、发展滞后的状况并未得到根本改变；发展方式仍然比较粗放，经济增长的质量和效益不高；工业基础仍然薄弱，主导产业不突出，产业优势不明显，企业规模偏小，竞争力不强，发展后劲不足；县域经济整体实力较弱，发展不平衡；财政增收压力大，收支矛盾突出，政府性债务较重；社会保障体系还不够完善，城乡居民收入水平偏低；干部作风有待进一步改善，急躁冒进和消极懈怠的倾向同时存在；影响社会和谐稳定的矛盾和问题还比较突出，等等。这些困难和问题，有待于我们在今后的工作中高度重视并切实加以解决。

　　二、今后五年政府工作的主要任务

　　各位代表，刚刚闭幕的市第三次党代会描绘了今后五年抚州发展的宏伟蓝图，提出了鼓舞人心、催人奋进的目标任务。市政府将坚决贯彻落实好市党代会精神，在新的起点上，努力创造抚州发展的新辉煌。今后五年政府工作的总体要求是：高举中国特色社会主义伟大旗帜，以邓小平理论和“三个代表”重要思想为指导，以科学发展观为统领，以加快转变经济发展方式为主线，积极参与鄱阳湖生态经济区建设，主动融入海西经济区发展大局，全力打好工业强攻战、县域经济发展大会战、跑项争资攻坚战“三大战役”，推动经济社会又好又快发展，为建设殷实、文明、和谐的幸福抚州而不懈奋斗。

　　主要奋斗目标是：确保主要指标增幅略高于省“十二五”规划提出的目标，力争完成市“十二五”规划确定的各项目标任务。即：未来五年，全市生产总值年均增长12%以上，财政总收入年均增长18%以上，全社会固定资产投资年均增长22%以上，规模以上工业增加值年均增长24%以上，社会消费品零售总额年均增长16%以上，出口总额年均增长15%以上，实际利用外资年均增长10%以上，城镇人均可支配收入和农民人均纯收入年均分别增长11%以上。

　　完成上述目标任务，我们要抓好以下几方面的重点工作：

　　（一）全力打好“三大战役”，稳步壮大经济实力

　　打好工业强攻战。以园区为主战场，以产业为依托，以企业为抓手，集中时间、集中力量，打一场工业强攻战。力争全市规模以上工业增加值三年翻一番，到2013年，达到320亿元以上。到2015年，全市工业实力明显提升，产业竞争力明显增强，初步建成新型工业化体系。一是完善园区功能，提升承载能力。进一步加大各级财政对工业园区的“输血”支持力度，重点投向园区提质、扩容，包括水、电、路、通信、污水管网等基础设施，物流、金融、商业等配套设施，以及公交、公园、员工公寓、活动中心等公共设施的建设。积极支持有条件的园区申报省重点项目和省产业基地，争取用地指标，保障重点项目用地；实行最严格的用地制度，开展存量土地清理、盘活、挖潜，提高园区建筑容积率、土地投资强度和土地综合利用率，实现节约用地和集约用地。二是注重特色兴工，培育支柱产业。明确全市各工业园区的产业特色及功能定位，围绕生物医药、纺织服装、机电汽车、食品加工、化工建材、有色金属加工六大主导产业，做大做强金巢生物医药、临川有色金属加工、金溪香料、崇仁变电设备、东乡变性淀粉、宜黄塑料、黎川日用耐热陶瓷和鞋业、南城南丰农副产品加工、广昌食品加工等特色园区和产业基地。实行差异化发展和品牌发展战略，改造提升传统产业，培育发展新兴产业，努力将主导产业培育成为附加值高、带动力强的支柱产业，着力打造一批在全省有影响力、有竞争力的特色化、专业化园区。三是突出龙头带动，扶持骨干企业。集中土地、电力、劳力、财力等资源要素，向优势、骨干企业倾斜，鼓励企业科技创新、靠大联强、兼并重组和改制上市，重点扶持江铃底盘、燕京惠泉啤酒、博雅生物制药、添光化工、珍视明药业、康舒陶瓷、明恒纺织、江西变电、恒兴食品等一批创税能力强、市场前景好的企业做大做强，形成示范带动效应。

　　打好县域经济发展大会战。县域经济是全市经济大厦的基石，今后五年内，要高度重视和大力推动县域经济发展，力争各县（区）生产总值、财政总收入、规模以上工业增加值、固定资产投资等主要经济指标实现翻番，县域经济综合实力明显增强。一是突出县域特色。各县（区）要因地制宜，突出特色，扬优成势，努力做到“工业一县一业，农业一县一品，三产全面发展”。工业方面，支持推动各县（区）选择确定1—2个主导产业重点培育、加快发展。农业方面，实行区域化布局、标准化生产，提高农产品精深加工水平。第三产业方面，以市场建设为重点，加快现代物流、现代旅游、金融保险、信息咨询等产业发展。二是改革体制机制。为县（区）放权松绑，形成有利于县域发展的充满活力的体制机制。在法律法规许可范围内，能下放的权限全部下放，能授权的尽量授权，给予县（区）更大的发展空间和更多的自主权。特别要理顺好临川区和金巢经济开发区的体制机制。市政府各部门、各单位应本着多服务不干预、多帮忙不添乱、多设路标不设路障的原则，促进县域经济快速发展，使县域经济在加快抚州发展中挑重担、扛大梁。三是完善考核体系。按照“又好又快”的要求，从侧重经济发展速度的考核转向发展速度、发展质量和发展后劲并重的考核。注重考核的导向性和科学性，从单一指标考核转向多元指标考核。在目标上，实现生产总值、财政收入、规模以上工业增加值、固定资产投资等重大指标的高效率增长；在内容上，实行“五比”，即纵比“增幅”、横比“份额”、内比“人均”、外比“排位”、综比“质量”。

　　打好跑项争资攻坚战。项目是加快发展的关键。要将争取国家和省的项目、资金作为持续增强有效投入的重要抓手，切实加大跑项目争资金力度。一是超前谋划开发项目。按照“前期储备一批、建设实施一批、超前谋划一批”的思路，做好项目谋划开发工作。确保市级项目储备库项目规模超过300亿元，常年保有量在150个以上，做到随时可拿出一批项目向上申报、对外招商。各县（区）也要注重建设和扩充项目储备库。二是把握重点争取项目。围绕国家重大项目、重大产业和重大政策，积极开展工作，力争我市有更多的重大项目进入国家和省相关规划，得到国家和省的审批。当前，重点要抢抓中央1号文件和中央水利工作会议带来的新机遇，加大跑项争资力度，促进我市水利事业实现新的跨越。三是加强衔接推进项目。积极推进廖坊灌区二期等工程建设，支持配合向莆铁路、杭南长客专、抚吉高速公路建设，确保项目如期建成通车；加快推进鹰梅铁路、衡茶吉铁路东延前期工作，争取国家发改委尽快批复鹰梅铁路项目立项，力争衡茶吉铁路东延项目列入国家“十二五”铁路网规划；按照国家民航发展“十二五”规划要求，积极推进抚州机场前期工作。力争大唐抚州电厂今年年底前获国家发改委核准，明年上半年正式开工建设，2013年年底前1台100万千瓦机组建成投产。四是广融资金支撑项目。在积极向上争取资金、严管财政资金的同时，还要善引外商资本、广融信贷资本、激活民间资本，为项目建设提供可靠的资金支持。

　　（二）大力发展现代农业，巩固农业基础地位

　　大力发展特色农业。一是实施特色强农战略。着力建设优势突出和特色鲜明的产业带和主产区，重点做大做强做优南丰蜜桔、广昌白莲、崇仁麻鸡、东乡生猪、黎川食用菌、资溪白茶、临川西瓜等特色优势产业，到2015年，全市特色农业占农业产值的比重提高到60%以上。二是调优农业结构。加大政策、资金引导力度，扩大特色经济作物和特色畜禽、水产品养殖，努力打造一批富有特色的种植、养殖专业乡镇和专业村组，大力发展专业合作社，促进农业增效、农民增收。

　　大力发展规模农业。一是扩大种养规模。重点建设优质稻、生猪、蔬菜、烤烟、水产养殖、油茶、毛竹、中药材、花卉苗木等农业产业基地。确保粮食播种面积稳定在610万亩以上，总产在260万吨以上。力争到2015年，全市果业种植面积达到140万亩，蔬菜种植面积达到120万亩，畜禽规模养殖比重达到83％。二是扶持龙头企业。五年新增市级以上农业产业化龙头企业100家，其中，主营业务收入超亿元的14家以上，超10亿元的2家以上。新增年交易额超亿元专业市场2个。三是提高农业机械化水平。保持全市农机总动力年均增长12%以上，到2015年突破710万千瓦。

　　大力发展生态农业。一是建设农业生态示范基地。充分发挥我市的生态优势，在抓好现有农业生态示范园区建设的基础上，新规划建设一批高标准的示范基地和绿色有机农产品生产基地。鼓励有条件的地区发展全区域的有机农产品生产。二是培育和保护生态农业品牌。加大“三品一标”认证工作力度。力争五年内新增无公害农产品80个，绿色（有机）农产品40个，省级名牌农产品10个，农业部农产品地理标志登记保护产品5个。加强品牌保护，强化证后监管，确保品牌的公信力和美誉度。三是发展新型农业形态。积极引导生态农业向观光、休闲、体验等方向拓展，延伸产业链条。

　　大力发展安全农业。一是完善农产品质量安全检测体系。建设一批乡镇质检站，增设一批农药残留速测点。以蔬菜为重点，建立健全农产品市场准入制度和追溯体系，强化农产品从农田到餐桌的全程监管。二是推进农业标准化建设。抓好蔬菜、茶叶、水果标准园和畜禽养殖标准示范场、水产健康养殖场等“三园两场”示范平台建设。充分发挥“三品一标”在制度规范、技术标准等方面的优势，带动和引领整个农产品标准化生产、全程质量控制。三是强化动植物疫情防控。确保农作物重大病虫害统防统治覆盖率达到65%以上，专业化防治达到35%以上，重大动物疫情总体保持平稳。

　　（三）繁荣发展现代服务业，培植扩大竞争优势

　　立足资源优势，大力发展现代旅游业。管理、开发和利用好我市独特的自然景观和人文景观，明确全市的旅游定位，打响和做大做强特色旅游“三大品牌”：依托独具特色的临川名人文化、戏曲文化、民俗文化、宗教文化、红色文化及建筑遗存等资源，打响“文化游”品牌；依托良好的生态环境，丰富的森林、湖泊、湿地等资源，打响“生态游”品牌；依托日趋便利的交通条件，日益凸显的区位优势，优质的温泉、农家乐项目等资源，打响“休闲游”品牌。一是加大项目开发建设力度。摸清全市旅游资源，完善旅游发展规划，建立、充实旅游项目库和风景名胜资源库，不断完善提升一批、建设发展一批、规划推动一批重点旅游景区和重点旅游项目。二是创新旅游管理体制。鼓励旅游景区所有权、经营权、开发权分离，支持条件成熟的景区组建景区管委会。三是加强区域合作。将我市精品景区与鄱阳湖生态旅游示范区的三大旅游圈、九大旅游区和海西旅游区各条精品线路紧密相联，形成圈圈交织、有机组合，提升我市旅游项目的吸引力。四是搞好宣传营销。加大媒体宣传力度，积极参加各类旅游促销活动，大力推介大觉山等旅游景区，积极开展旅游强县、A级景区、自然保护区、星级饭店、省级和国家级风景名胜区等旅游品牌创建活动，支持和推动拟岘台、名人雕塑园、临川日月温泉、南丰傅坊温泉、崇仁汤溪温泉、乐安流坑、宜黄曹山景区、金溪竹桥古村、南城麻姑山景区、黎川洲湖景区、东乡佛岭风景区、广昌红色雕塑园和资溪法水温泉等一批景区创建A级景区。力争到2015年，全市旅游综合收入达到GDP的10%左右，我市成为全省旅游强市，资溪、临川、乐安、南城、南丰等县成为全省旅游强县，建成8个以上4A级景区。

　　依托区位交通优势，积极发展现代物流业。一是建设物流基础设施。以市城区为中心，以南城、南丰、广昌等物流大县为重点，建设一批市、县（区）物流中心和物流园区，把抚州建成全省五大物流服务大通道之一。依托向莆铁路，积极推动抚州至九江城西港水运口岸通道、南昌至抚州至福州港通道建设和抚州北站铁路口岸作业区建设；加强与福建莆田合作，推进抚州“无水港”和国际集装箱货场建设，实现“铁公海”联运。二是培植物流龙头企业。将发展第三方物流作为今后物流企业组织创新的重点，进一步放开物流市场，培育壮大物流企业实力，重点打造2个以上在全省、全国有影响的物流龙头企业，提高市场竞争力。三是打造物流信息平台。构建物联网应用基础框架，开展物联网应用技术研发，着力打造开放、标准、高效的市级物流公共信息平台。

　　强化政策引导，促进金融业发展。一是优化金融生态环境。加快社会信用体系建设，建立以中小企业为重点、覆盖经济社会各个方面的信用数据库，为扩大贷款提供信息支撑和制度保障。大力推广“信用共同体+征信+金融服务”、“农村金融服务中心”等农村信用体系示范点建设模式，以及“信用培植+信贷工厂”等中小企业信用试验区建设模式，降低企业融资成本，减少金融机构放贷风险。积极推动企业外部信用评级工作，加大征信产品在社会经济交往中的应用。积极构筑政银企对接平台，引导信贷资金向“三农”和中小企业倾斜，满足实体经济发展需求。二是完善金融服务体系。加大市外、省外金融机构引进力度，加快抚州农村商业银行、村镇银行和小额贷款公司组建步伐，做大做强融资性担保机构，千方百计做大信贷总量，不断提高我市贷款投放占全省的比重。三是拓宽融资渠道。积极探索中小企业集合票据、集合信托、短期融资券等新型融资方式，为企业发展提供强有力的资金支持。

　　发挥潜在优势，带动相关服务业发展。充分发挥抚州教育品牌优势，大力发展为教育服务的产业，实现“留学生现象”到“留学生经济”的跨越。扩大中学生“留学效应”，带动更多的学生、家长和家庭到抚州生活消费、经商办厂、投资置业，通过人口和资金的加速涌入，带动为教育服务的房地产、商贸、餐饮、金融、家政、交通运输等产业蓬勃发展，使教育成为引领现代服务业发展、拉动我市经济增长的强大引擎。发挥“临川建筑”品牌优势，大力发展抚州的建筑业，使之成为强市富民的重要产业。支持和规范房地产业发展，并以此带动住宅装修、家用电器、家具、建筑等产业的发展，使房地产业成为拉动内需、加快城镇化进程的重要力量。与此同时，大力发展工程设计、文化创意、信息咨询和科技服务等生产性服务业，积极拓展职业培训、医疗保健、休闲娱乐、养老护理等新兴服务业，改造提升商贸、社区服务、餐饮等传统服务业，大力引进大型品牌连锁商家，促进服务业拓宽领域、扩大规模、优化结构、提升层次。

　　（四）加快推进新型城镇化，统筹城乡发展

　　以人为本，全面改善人居环境，努力建设人民满意的幸福城市。以城带乡，进一步加快新型城镇化步伐，力争全市城镇化率每年提高2个百分点以上，到2015年，市中心城区人口达到60万人以上。

　　做大做强市中心城区。一是严格执行规划。坚持规划引领，高起点、高标准做好城市规划设计，做到先规划、后建设，无规划、不建设。严格规划管理。对新、老城区以及金巢经济开发区的规划，实行集中统一管理，切实维护规划的严肃性。严格规划执法，重拳出击，严厉整治违法用地、违法建设“两违”行为。二是完善城市功能。坚持一手抓新城区建设，一手抓旧城改造。按照适度超前的原则，搞好城市基础设施建设尤其是功能性基础设施建设。在与城市规模、经济社会发展水平相适应的前提下，重点规划、建设和改造一批布局合理、关系民生、承载城市经济社会发展需要的公共服务配套项目，为市民出行入住、工作生活创造良好条件。加快推进城南新区建设，着力打造生活便利、交通便捷、商贸繁荣的城市新区。大力推进棚户区、小街小巷以及老居民区的改造，让老城区居民分享城市建设的成果。三是严格项目监管。做到严把“三关”，即：严把立项程序关，严格执行政府投资项目基本建设程序，实行先论证、后审批、再实施。严把资金监管关，坚持概算控制预算、预算控制决算的原则，防止和杜绝突破建设规模超预算投入。严格执行项目资金评审和决算审计程序，堵塞资金漏洞，防止资金浪费，降低工程建设成本。严把工程质量关，实行项目法人责任制、合同管理制、招投标制和工程监理制，确保工程质量。四是加强城市管理。按照“横向到边、纵向到底”的要求，完善“两级政府、三级管理、四级网络”体制，进一步加强小街小巷、摆摊设点、城市保洁、车辆停放、交通秩序等薄弱环节的整治管理，实现无缝隙全覆盖、无空档全天候管理。整合城市信息化资源，推进城管、人防、应急“三网一库”建设，建立“大城管”格局。

　　加快推进县城和小城镇建设。立足各县城的区位条件、资源禀赋、产业特色、人口规模等因素，拓展县城规模，完善基础设施，优化城市形态，提升县城承载工业、繁荣三产、拉动农业升级和农民转移的功能。促进东乡、南城等有条件的县加速向中等城市目标迈进。坚持统一规划、分步实施，加强2个省级示范镇、21个市级示范镇的水、电、路、垃圾处理等基础设施建设，完善学校、幼儿园、卫生院、便民服务中心等公益设施，切实提高示范镇的承载能力和保障能力。加强政策引导,建立财政资金、社会资金、信贷资金多元化投入体制机制，促进示范镇建设和发展。通过示范镇建设，以点带面，统筹推进全市小城镇建设，提高城镇化水平。

　　扎实推进新农村建设。一是建设新村镇。围绕村镇联动、大村改造、移民搬迁、古村整治及沿主干道、主流域、区域边际“三沿建设”等重点，每年确定1000个左右新农村点，落实建设资金，编制村庄规划，启动村点建设。二是发展新产业。以市场为导向，结合各个新农村建设点产业基础和资源优势，制定产业发展规划，落实到每家每户，努力形成“一村一品”。三是培育新农民。积极为建设点农民量身打造就业、创业方面的技能培训项目，拓宽就业和创业渠道，培养能生产、会经营的新型农民。四是形成新风尚。繁荣农村文化，丰富群众生活，提高群众的科技文化素质和道德素养，增强群众的遵纪守法意识，形成和谐文明、积极向上的村风民风。

　　（五）进一步深化改革扩大开放，不断提升区域发展竞争力

　　主动参与鄱阳湖生态经济区和海西经济区建设。一是加快推进昌抚经济一体化。加强与南昌的基础设施对接，大力发展各类专业市场，促进两地间人流、物流、资金流等要素流动。实施与南昌差异化发展战略，与南昌形成协作互补、错位发展的格局。充分发挥和扩大抚州的生态文化优势，努力将抚州建成南昌和海西经济区的休闲、旅游“后花园”。二是加快承接闽台地区产业梯度转移。沿向莆铁路、福银高速、济广高速和鹰梅铁路展开生产力布局，打造以闽台投资为主体的新兴工业走廊，尽快形成闽台资企业的聚集地。加强与闽台特别是台湾的农业合作，提高我市农业现代化水平。三是加快对接重大基础设施项目。积极推进抚吉高速及其延伸段、资溪至福建光泽、船顶隘至广昌等高速公路建设，实现“县县通高速”目标，努力把抚州建设成为连接海西与内陆地区的交通大通道，面向沿海地区的产业梯度转移承接区、优质农产品生产加工集散区和文化生态旅游观光休闲区。

　　积极发展开放型经济。一是大力开展招商引资。围绕六大主导产业和六个特色产业基地，有针对性地开展招商活动。力争在引进国内500强和全球500强等大企业上取得新的突破。落实亲商、安商、富商的各项举措，发挥已落户企业的积极性，引进上下游合作配套企业，尽快形成产业集群。二是努力扩大对外出口。壮大出口经营队伍，着力培植一批主业突出、核心竞争力强、年出口额超3000万美元的出口龙头企业，引进和培育一批生产型出口企业。积极抢抓国家加工贸易政策重大调整、沿海地区加工贸易制造业加速向中西部地区转移的机遇，发展加工贸易。三是加快搭建发展平台。积极推动在抚州设立海关和商检机构，进一步降低企业报关报检和通关成本，优化投资环境，增强区域经济的吸引力和竞争力。

　　深入推进重点领域各项改革。深化财政管理体制改革，推进部门预算管理改革，继续完善国库集中支付、乡财县管和村账乡代理制度。深化医药卫生体制改革，努力建立起较为完善的基本医疗保障、基本药物制度、基本医疗服务和基本公共卫生服务体系，抓好公立医院改革。加快文化体制改革。通过整合行业资源，共同投资，相互参股，跨地区、跨行业兼并和联合重组等方式，形成1—2个具有较强竞争力和影响力的大型文化企业或文化产业集团。推动事业单位人事制度改革，深化收入分配制度改革。推进产权制度和要素市场改革。开展农村综合改革，完善集体林权制度配套改革，推进国有林场改革和小型水利基础设施产权制度改革。开展生态环保改革试点，深化资源环境有偿使用制度改革。

　　大力推进全民创业。一是培育创业主体。鼓励和引导在外务工、经商和创办企业的成功人士回乡创业，支持帮助本地个体工商业主和本土企业家“二次创业”。鼓励和引导大中专毕业生回抚创业，尤其是鼓励和支持科研人员利用科研成果创办、领办企业或通过技术入股等形式参与创业。培育壮大各种行业产业协会、经济联合体及各类中介服务组织，扶持引导经纪人队伍创业。二是完善创业政策。落实好扶持创业的各项政策措施，搭建创业政策、孵化、融资、培训、信息等平台，着力打造全民创业的“成本洼地”和“政策高地”。三是加强创业引导。积极鼓励和引导创业者围绕工业特色产业，发展上下游关联配套企业。积极引导各类创业主体围绕农业特色产业，参与种养、加工、销售、服务等行业。鼓励和支持民间资本参与文化、旅游产业的开发建设。四是优化创业环境。大力弘扬创业精神，使创业成为全社会共同的价值取向。进一步转变政府职能，加强行政服务中心建设，优化“窗口”服务，打造最优的创业环境。

　　（六）全面加强民生建设，维护社会和谐稳定

　　加强和创新社会管理。广泛动员和组织群众依法参与社会管理，发挥社会组织的积极作用，完善社会管理格局。培育扶持和依法管理社会组织，支持、引导其参与社会管理和服务。加强信访、人民调解、行政调解、司法调解工作，拓宽社情民意表达渠道，切实解决好群众反映强烈的问题。健全突发事件应急体制，提高全社会危机管理和抗风险能力。进一步加强政府网站建设，推进政府信息公开，建立健全及时、准确、透明的信息公开机制，正确引导和应对社会舆论特别是网络舆情。加强社会治安综合治理，严密防范、依法打击各类违法犯罪活动，切实维护社会公平正义，切实维护法制的尊严，切实维护社会治安持续稳定。落实企业安全生产和产品质量主体责任，加强隐患排查、专项整治、标准化建设、打非治违、应急救援等工作，坚决遏制重特大安全生产事故发生。进一步强化食品药品监督工作，保障群众饮食和用药安全。

　　着力健全社会保障体系。不断提高人民群众生活质量，努力实现居民收入增长和经济发展基本同步。以基本养老、基本医疗、最低生活保障制度为重点，以发展社会保险为主、慈善事业为补充，不断扩大社会保障覆盖面，逐步提高社会保障水平，“十二五”期间基本实现社会保险制度全覆盖，努力实现人人享有基本生活保障的目标。进一步完善城镇职工和居民基本医疗保险、新型农村合作医疗制度、大病救助制度，不断提高医疗保障水平。加快构建以廉租房、公租房为主要形式的保障性住房体系，加快棚户区改造步伐，力争“十二五”期间基本解决全市低收入住房困难家庭的住房问题。建立健全以居家养老为基础、社区服务为依托、机构养老为补充的社会养老服务体系，重点抓好市中心城区老年公寓和县（区）中心敬老院建设，推进社会养老和居家养老项目建设。健全社会救助服务体系，切实提高城乡困难群众救助标准。加强自然灾害救助能力建设，加快推进减灾救灾指挥中心和救灾物资储备库建设，提高综合减灾水平。积极发展以扶老、助残、救孤、济困、赈灾为重点的社会福利事业，逐步拓展社会福利的保障范围。继续做好扶贫开发和深山、库区及地质灾害区移民工作。

　　大力发展各项社会事业。深入实施科技创新“六个一”工程，积极开展“科技入园”工作，加快科技成果向现实生产力转化。坚持教育优先发展战略，充分体现教育的公益性和普惠性，进一步打响抚州教育品牌，做大做强抚州教育，落实《抚州市中长期教育改革和发展规划纲要》的目标要求，稳步提高财政性教育经费支出比重，促进各级各类教育事业均衡协调发展。着力推进人才强市战略，加大引进、培养高层次人才工作力度，抢占加快发展的制高点。大力弘扬“临川文化”，高度重视历史文化遗存保护，加大对抚州古建筑的抢救、保护和利用,以及非物质文化遗产的发掘、保护和传承力度。大力发展医疗卫生事业，完善基层卫生服务体系，加快城乡公共卫生服务均等化步伐，提升卫生服务能力和水平，让人民群众得到更多的健康保障。加强生态环境保护，落实节能减排目标责任。加强人口计生工作，深化人口和计划生育综合改革，促进人口长期均衡发展。深入实施“全民健身计划”，进一步提升城乡居民的身体素质和健康水平。及时启动新一轮广播电视“村村通”工程建设，加快“数字影院”建设，进一步做好送电影下乡惠民工程。积极推进国防后备力量创新发展，切实做好征兵、武警反恐力量建设、现役军人随军家属就业、子女入学入托、干部士官转业安置等工作。

　　三、加强政府自身建设，切实提高执政能力和水平

　　抢抓新一轮发展机遇，对政府工作提出了新的更高的要求。全市各级政府和政府各部门，要围绕建设服务政府、法治政府、责任政府、廉洁政府，切实加强自身建设，提升工作水平，更好地为建设幸福抚州履职尽责。

　　（一）坚持为民执政。切实增强宗旨意识，关心群众疾苦、倾听群众呼声、了解群众意愿，想群众之所想，急群众之所急，解群众之所难，办群众之所需，千方百计为人民群众办好事、解难事，将更多的公共资源向民生倾斜，让人民群众更好地享受改革发展的成果。切实维护群众利益，想问题、作决策、办事情，首先考虑群众利益，坚决维护好绝大多数群众长远的、根本的利益，同时平衡好、协调好各种利益关系。切实改进干部作风，怀着对人民群众的深厚感情，从群众中来，到群众中去。用为人民群众办实事、解难事的实际效果，检验衡量政府工作效果。以公正公平、亲民爱民的实际行动，提高政府的公信力和人民群众的满意度。

　　（二）坚持依法行政。始终把依法行政贯穿到政府工作的全过程，严格按照法定程序、法定权限和法定职责，行使人民赋予的权力。坚持科学民主决策。进一步规范行政决策程序，完善行政决策风险评估机制，加强行政决策监督和责任追究，提高行政决策水平。自觉接受人大及其常委会的法律监督和工作监督，主动接受政协的民主监督，认真办理人大代表建议和政协委员提案，不断提高办理效率和质量。认真听取各民主党派、工商联、无党派人士、各人民团体及社会各界的意见和建议。高度重视新闻舆论和社会公众监督。全面推进政务公开，切实保障人民群众的知情权、参与权、表达权和监督权。

　　（三）坚持团结从政。切实维护全市团结和谐稳定的大局，切实维护市委的领导权威，团结依靠全市人民，努力营造风清、气正、劲足的创业干事氛围。坚持求真务实，始终做到察实情、说实话、办实事、求实效。坚持理论联系实际，主动深入基层、下到一线开展调查研究，在实践中理清发展思路，在实践中完善发展举措。坚持实事求是，一切从实际出发，尊重事实，把握规律，创造性地开展工作。力戒形式主义、劳民伤财，力戒虚报浮夸、急功近利，力戒弄虚作假、欺上瞒下。坚持真抓实干，把心思和精力放在抓工作、抓发展上，每项任务都做到有部署、有检查、有落实。注重工作实效，把工作成效作为衡量和检验工作优劣的最终标准。

　　（四）坚持廉洁勤政。切实落实党风廉政建设责任制，抓好政府系统党风廉政建设和反腐败工作，从严治政，廉洁从政。深入推进惩治和预防腐败体系建设，进一步加强国有资产管理、工程建设、公共资源交易、土地出让和政府采购等重点领域的监管，强化经济责任审计和重点项目审计，从源头上预防腐败，确保行政权力在阳光下规范运行。继续深化行政审批制度改革，规范和完善网上审批和电子监察系统建设，最大限度地压缩审批事项，简化办事程序，加快建立权责明晰、行为规范、运转协调、办事高效的行政运作机制。加强政风行风建设和机关效能建设，加强明查暗访，加大查处力度，下大力气整治办事拖拉、推诿扯皮、吃拿卡要等破坏形象、阻碍发展的现象，严厉查处乱收费、乱摊派、乱罚款、乱检查等增加企业和群众负担的行为，坚决惩治与民争利、以权谋私、贪污受贿等违纪违法行为，营造良好的发展环境。

　　各位代表，“雄关漫道真如铁，而今迈步从头越”。宏图已经绘就，号角已经吹响，站在新的历史起点上，面对新的机遇和挑战，面对全市人民的期待和重托，我们深感责任重大，使命光荣。让我们更加紧密地团结在以胡锦涛同志为总书记的党中央周围，高举邓小平理论和“三个代表”重要思想伟大旗帜，全面贯彻落实科学发展观，在省委、省政府和市委的坚强领导下，万众一心，开拓进取，为建设殷实、文明、和谐的幸福抚州而不懈奋斗！
